# Vertebral osteomyelitis in patients with an underlying malignancy or chronic kidney disease – who is at higher risk for adverse outcome?

**DOI:** 10.1007/s15010-024-02451-2

**Published:** 2024-12-21

**Authors:** Dorothee Jochimsen, Ayla Yagdiran, Charlotte Meyer-Schwickerath, Krishnan Sircar, Nikolaus Kernich, Peer Eysel, Carolyn Weber, Norma Jung

**Affiliations:** 1 Department I of Internal Medicine, Division of Infectious Diseases, University of Cologne, Cologne, Germany; 2 Department of Orthopedics and Trauma Surgery, University of Cologne, Cologne, Germany; 3 Department of Cardiothoracic Surgery, University of Cologne, Cologne, Germany

**Keywords:** Spondylodiscitis, Vertebral osteomyelitis, Treatment failure, 1-year-mortality, Recurrence, Malignant disease, Malignancy, Chronic kidney disease, Coagulase-negative staphylococci, CoNS, Staphylococcus aureus, S.aureus

## Abstract

**Purpose:**

Patients with vertebral osteomyelitis (VO) and comorbidities, notably chronic kidney disease (CKD), are at risk of early mortality. The aim of this study was to compare characteristics and outcomes of VO patients with an underlying malignancy (ONCO) to VO patients with CKD and VO patients without comorbidities (CONTROL).

**Methods:**

We performed a retrospective analysis of data which was prospectively collected between 2008 and 2020. Primary outcome was treatment failure defined as death and/or recurrence of VO within one year.

**Results:**

241 VO patients (ONCO = 56; CKD = 47; CONTROL = 138) were analysed. Treatment failure occurred in 26% of ONCO and 45% of CKD patients. *Staphylococcus aureus* was the most common causative pathogen in the CKD (57%) and CONTROL group (43%). ONCO patients showed a broader distribution of common VO-causing pathogens, with *coagulase-negative staphylococci* (CoNS) accounting for the highest proportion of causative bacteria (27%). Nevertheless, *S.aureus* was associated with a significantly higher risk of treatment failure in VO ONCO patients.

**Conclusion:**

Treatment failure in VO CKD patients was twice as high as in VO ONCO patients. However, both groups showed high treatment failure rates. CoNS should be considered when starting empirical antibiotic treatment in VO ONCO patients. Moreover, oncological patients with VO caused by *S.aureus* should be monitored closely.

## Introduction

Vertebral osteomyelitis (VO) is a rare but severe disease with a rising incidence [1–3]. Increasing age, multimorbidity, poorly controlled diabetes, renal failure, liver cirrhosis, malignancies, immunosuppression, invasive procedures, previous surgery and intravenous access devices are some of the most important risk factors causing VO [4–9]. Elderly people are particularly at risk of suffering from VO, mainly due to increasing comorbidities [9]. According to the Clinical Practice Guidelines of native VO by the Infectious Diseases Society of America (IDSA) from 2015 mortality ranges from 0–11% [10] whereas in recent studies reported 1-year-mortalities were even higher and reached around 20% [11–15]. High age and comorbidities, especially heart failure, liver failure and chronic kidney disease (CKD), [14, 16–19] are highly associated with an increased risk of mortality. Nevertheless, studies which specifically focus on one of these comorbid conditions in VO patients are scarce. End-stage renal disease is one comorbidity which is known to be associated with high mortality in VO patients. Overall-mortality in this patient group was 24% according to a systematic review from 2019 [20]*.* To our knowledge, so far there is no specific data regarding characteristics and outcome of VO patients with an underlying malignancy. However, due to immunosuppression, these patients might be at high risk of adverse outcome as well.

The aim of this study was to analyse VO patients with an underlying malignancy and to compare characteristics and outcomes to VO patients with CKD, a patient group with known adverse outcome, as well as to VO patients without any severe comorbidities who are expected to show good outcomes.

## Material and methods

### Patient selection

**Fig. 1:**
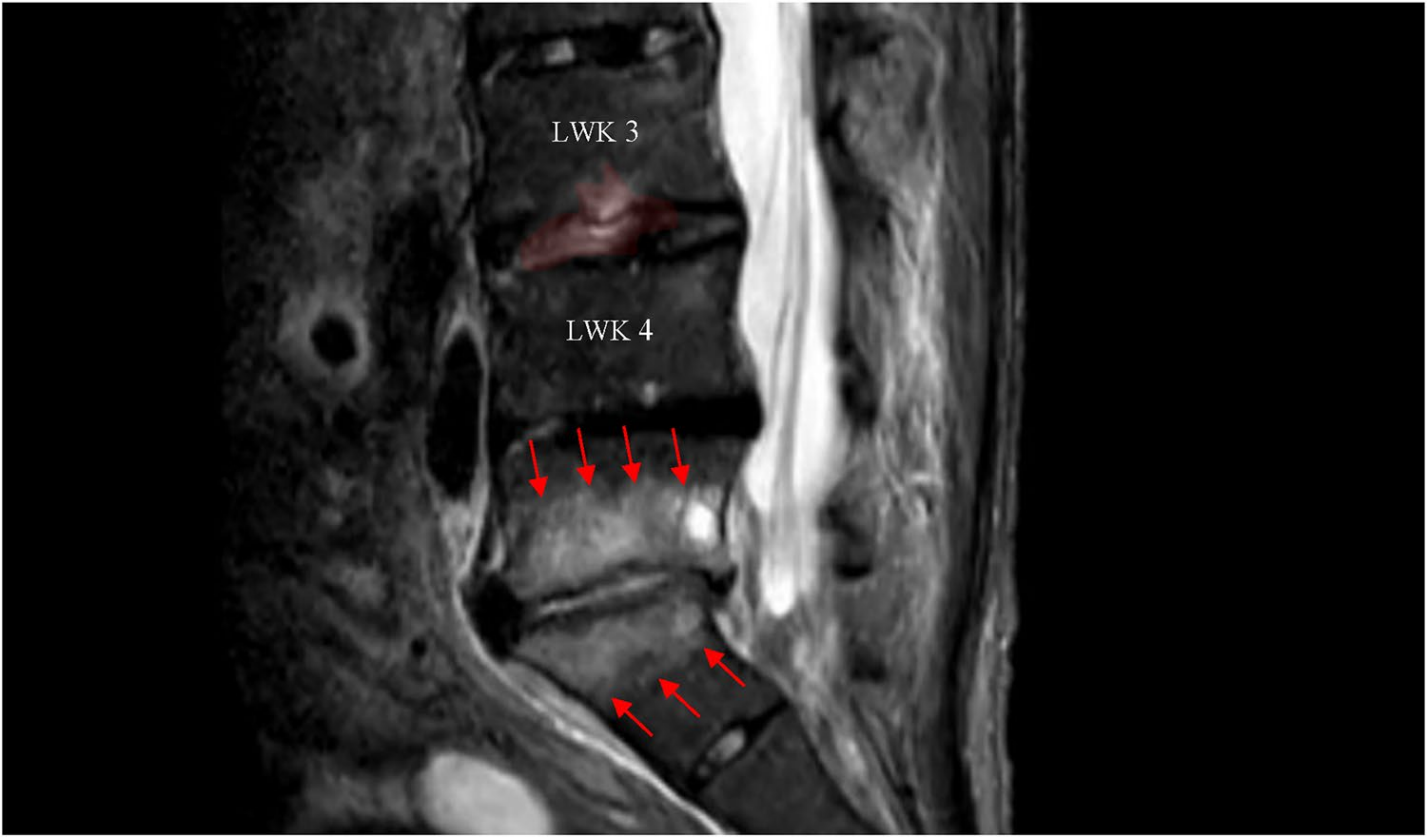
Characteristic MRI of VO in L3/4 and L5/S1. MRI of the lumbar spine of a patient presenting to the emergency department with fever, severe back pain, and elevated inflammatory markers (C-reactive protein 74 mg/L, leukocytes 12.000/µL). On STIR sequences, there was signal enhancement in the intervertebral disc and the superior and inferior endplates of the L5/S1 motion segment, accompanied by corresponding contrast enhancement (highlighted with arrows). Additionally, there was mild contrast uptake in the adjacent paravertebral soft tissues. In light of the clinical presentation, VO was strongly suspected in the L5/S1 segment. Minor changes were also observed in the L3/L4 segment (highlighted in red colour), where early inflammatory changes were also possible. *Enterococcus faecalis* was detected in blood cultures and in the intraoperative samples of the L3/4 and L5/S1 discs and verified the diagnosis of VO

Data were used from the former European “Spine Tango” database and the German “Deutsche Wirbelsäulen Gesellschaft (DWG)” register from 2008 until 2020. VO was diagnosed by an orthopaedic surgeon and confirmed by an infectious disease specialist at the Department for Orthopaedics and Trauma at a tertiary referral hospital based on clinical findings (characteristic new or worsening back or leg pain and/or new neurologic symptoms), radiological findings (characteristic magnetic resonance imaging or an abscess or vertebral body destruction detected by computed tomography) and microbiological as well as histopathological findings (blood cultures and/or vertebral biopsy or abscess biopsy) if a pathogen was identified (Fig. 1). The relevance and pathogenicity of the microbiological findings were assessed by an infectious disease specialist. If a virulent pathogen, such as *Staphylococcus aureus* or gram-negative bacteria, was identified in one or more samples, it was considered the causative pathogen. If low-virulence organisms were detected, such as coagulase-negative staphylococci or *Propionibacterium* species, they were considered clinically significant if found in two or more samples. In case no pathogen could be identified and laboratory values were normal at onset although no previous antimicrobial treatment was administered, the diagnosis was rejected, particularly if a more plausible alternative diagnosis, such as a tumour or erosive osteochondrosis, was suggested by histopathological findings. Information about comorbidities was retrospectively collected from the medical history file. Patients who were included in the ONCO group had been diagnosed with either a haematologic malignancy or a solid tumour. If patients with a malignancy suffered from CKD at the same time and vice versa, they were excluded from the analysis. The CONTROL group was defined as VO patients with no history of CKD, malignancy, diabetes, chronic obstructive pulmonary disease (COPD), inflammatory bowel disease (IBD), rheumatic disease, immunosuppression, or heart failure.

Follow-up was at least 1 year. The causing pathogen of VO was identified by blood cultures and/or intraoperative samples and treated with targeted antimicrobial therapy. If no pathogen could be detected, empirical antimicrobial therapy was administered with the local standard regimen (ceftriaxone and flucloxacillin) for 2 weeks intravenously, followed by a highly bioavailable oral antimicrobial therapy. The total duration of antimicrobial treatment differed between 6 to 12 weeks depending on the individual severity of the disease and risk of treatment failure (e.g., foreign-material associated).

### Data collection

The following data were used in our analysis: age, sex, body mass index, bacteraemia, identified pathogen (characterized in groups), neurological deficits, psoas abscess, empyema, infectious endocarditis (IE), laboratory values, American Society of Anaesthesiologists (ASA) Physical Status Classification System to classify the patients’ comorbidities (ASA Class I is defined as a normal healthy patient and Class V as a moribund patient not expected to survive without surgery), previous surgical procedures on the spine, surgical treatment, days until surgery, length of hospital stay, 1-year-mortality and recurrence of disease. Besides malignancy and CKD, the following comorbidities were separately listed: diabetes, COPD, IBD, rheumatic disease, heart failure, alcohol and drug abuse. In addition, comorbidities were categorized according to severity using the Charlson comorbidity index (CCI). The CCI score classifies comorbidities into mild (CCI 1–2), moderate (CCI 3–4), and severe (CCI > 5) and estimates the 1-year mortality of patients.

### Definition of endpoints

The endpoint of our study was treatment failure defined as death and/or recurrence of the disease within one year.

### Statistical analysis

Unless otherwise stated, continuous variables are described according to the normal distribution with mean ± standard deviation or median (interquartile range) and compared with an unpaired *t*-test or Mann–Whitney *U*-test. Discrete variables are expressed as percentages and tested with Pearson’s chi-square test or, if validity conditions were not met, Fisher’s exact test. Missing data were not imputed and were assumed to be missing at random. Potential risk factors for the combined end point consisting of 1-year mortality and/or recurrence were determined using logistic regression. After univariable analysis, all variables with a p value less than 0.05 were included in the multivariable model using forward selection (likelihood ratio, p_in_ = 0.05). Results are presented as odds ratios with corresponding 95% confidence intervals and p-values. The log-rank test was used to test for differences in long-term mortality. The resulting p-values and additional Kaplan–Meier survival curves are shown here. All p-values reported are 2-sided. Statistical analyses were performed using SPSS Statistics version 28.

### Ethics

Every patient included in the register was at least 18 years old. Ethical approval was given by the Faculty of Medicine at the University of Cologne (09-182) and complies with the principles of the Declaration of Helsinki (1996). All patients gave written informed consent for participation in the trial.

## Results

In total, 241 of the 355 VO patients of the DWG register were included in our study. We enrolled 56 patients with the diagnosis of a malignant disease (VO ONCO patients), 47 patients with CKD (VO CKD patients) and 138 patients without comorbidities (VO CONTROL group) in the analysis. In the ONCO group, 45 patients were diagnosed with a solid tumour, 8 with a haematologic malignancy and 3 patients had both, solid as well as haematologic cancer.

### Baseline characteristics

Demographics and clinical characteristics are shown in Table 1. P-values refer to the comparison of ONCO patients versus CONTROL patients and CKD patients versus CONTROL patients. VO occurred predominantly in men and affected particularly elderly patients. The mean age of patients in the CONTROL group was 68 years [57.8–74.3]. In contrast, the mean age of patients in the ONCO group (71 [63.8–77.0] years, P = 0.037) and CKD group (73 [61.8–79.3] years, P = 0.012) was significantly higher. 67.9% of VO ONCO patients were male. Most patients of the ONCO group (67.9%, P = 0.001) and the CKD group (74.5%, P = 0.001) were categorized as severely ill with a CCI score ≥ 5 which differed significantly from the CONTROL group (10.1%). Predominant comorbidities in the VO ONCO group were diabetes (17.9%), heart failure (16.1%) and COPD (10.7%). In the CKD group, there was a strikingly high percentage of patients with heart failure (51.1%) and diabetes (46.8%) as secondary diagnoses. Bacteraemia was present in only 30.4% of the ONCO group (P = 0.618). In CKD patients, bacteraemia occurred in half of the patients and was significantly more frequent than in the CONTROL group (44.7% vs. 26.8%, P = 0.023). Regarding the bacterial species that could be identified as underlying pathogen, *Staphylococcus aureus* was the most common causative pathogen in the CKD (56.7%) and CONTROL group (42.6%). In the ONCO group, *S.aureus* was identified significantly less frequently (22.7%, P = 0.024). The most common causative pathogens in the ONCO group were *coagulase-negative staphylococci* (CoNS) (27.3%), followed by *S.aureus* (22.7%), gram-negative species (15.9%) and *streptococci* (11.4%). *Candida species* could be exclusively found in the ONCO group (4.5%, P = 0.031).

**Table 1:** Baseline characteristics, clinical data and outcome of vertebral osteomyelitis (VO) patients without comorbidities (CONTROL), VO patients with an underlying malignancy (ONCO) and VO patients with CKD

Patient group n = 241	CONTROL n = 138, n (%)	ONCO n = 56, n (%)	*P*	CKD n = 47, n (%)	*P*
**Age** (years)	68.0 [57.8–74.3]	71.0 [63.8–77.0]	0.037*	73.0 [61.8–79.3]	0.012*
**Male**	89/138 (64.5)	38/56 (67.9)	0.654	36/47 (76.6)	0.126
**Female**	49/138 (35.5)	18/56 (32.1)	0.654	11/47 (23.4)	0.126
**BMI** (kg/m^2^)	26.1 [23.4–29.3]	23.4 [21.2–26.4]	0.001*	27.2 [22.0–31.6]	0.585
**Charlson Comorbidity Index (CCI)**					
Mild (≤ 2)	65/138 (47.1)	7/56 (12.5)	0.001*	3/47 (6.4)	0.001*
Moderate (3–4)	59/138 (42.8)	11/56 (19.6)	0.002*	9/47 (19.1)	0.004*
Severe (≥ 5)	14/138 (10.1)	38/56 (67.9)	0.001*	35/47 (74.5)	0.001*
**Number of vertebral bodies affected**					
1 segment	113/138 (81.9)	40/56 (71.4)	0.106	40/47 (85.1)	0.614
2 segments	21/138 (15.2)	13/56 (23.2)	0.184	5/47 (10.6)	0.435
≥ 2 segments	4/138 (2.9%)	3/56 (5.4%)	0.422	2/47 (4.3)	0.659
**Spinal region affected**					
cervical	3/138 (2.2)	1/56 (1.8)	0.861	3/47 (6.4)	0.189
thoracic	25/138 (18.1)	14/56 (25.0)	0.278	11/47 (23.4)	0.429
thoracolumbar	7/138 (5.1)	0/56 (0)	0.027*	6/47 (12.8)	0.093
lumbar	88/138 (63.8)	33/56 (58.9)	0.528	23/47 (48.9)	0.073
lumbosacral	11/138 (8.0)	3/56 (5.4)	0.512	3/47 (6.4)	0.718
multifocal	4/138 (2.9)	5/56 (8.9)	0.087	1/47 (2.1)	0.773
**ASA**					
1	11/134 (8.2)	1/51 (2.0)	0.086	0/45 (0)	0.010*
2	58/134 (43.3)	9/51 (17.6)	0.001*	5/45 (11.1)	0.001*
3	58/134 (43.3)	35/51 (68.6)	0.002*	33/45 (73.3)	0.001*
4	7/134 (5.2)	6/51 (11.8)	0.138	7/45 (15.6)	0.037*
**Comorbidities** (yes)					
Diabetes	0/138 (0)	10/56 (17.9)	0.001*	22/47 (46.8)	0.001*
COPD	0/138 (0)	6/56 (10.7)	0.001*	4/47 (8.5)	0.001*
IBD	0/138 (0)	0/56 (0)	-	0/47 (0)	-
Rheumatic disease	0/138 (0)	3/56 (5.4)	0.006*	3/47 (6.4)	0.004*
Heart failure	0/138 (0)	9/56 (16.1)	0.001*	24/47 (51.1)	0.001*
Alcohol abuse	8/138 (5.8)	2/56 (3.6)	0.511	7/47 (14.9)	0.063
Drug abuse	5/138 (3.6)	1/56 (1.8)	0.480	1/47 (2.1)	0.601
Bacteraemia	37/138 (26.8)	17/56 (30.4)	0.618	21/47 (44.7)	0.023*
**Manifestations** (yes)					
IE	4/138 (2.9)	2/56 (3.6)	0.809	4/47 (8.5)	0.127
Neurological deficit	23/138 (16.7)	8/56 (14.3)	0.682	15/47 (31.9)	0.025*
Psoas abscess	29/138 (21)	13/56 (23.3)	0.736	11/47 (23.4)	0.731
Empyema	46/138 (33.3)	19/56 (33.9)	0.937	12/47 (25.6%)	0.319
**Laboratory values at T0**					
CRP (mg/l)	52.3[23.3—113.2]	60.1 [27.4—106.9]	0.598	100.3 [33.4–161.4]	0.031*
Leukocytes (× 10^9^/l)	8.9 [7.2–11.0]	9.0 [6.2–11.4]	0.530	8.5 [7.0–11.7]	0.952
Hb (g/dl)	11.8 ± 1.9	10.9 ± 2.0	0.643	10.4 ± 2.1	0.001*
Creatinine (mg/dl)	0.8 [0.6–1.0]	0.8 [0.7–1.3]	0.026*	1.7 [1.2–3.0]	0.001*
GFR (ml/min)	88.0 [72.0–114.0]	76.5 [51.0–98.0]	0.007*	37.0 [29.0–57.0]	0.001*
**Bacterial species**					
*S.aureus*	40/94 (42.6)	10/44 (22.7)	0.024*	17/30 (56.7)	0.177
*CoNS*	18/94 (19.1)	12/44 (27.3)	0.281	5/30 (16.7)	0.761
*Gram-negative spp.*	8/94 (8.5)	7/44 (15.9)	0.205	2/30 (6.7)	0.742
*Streptococcus spp.*	4/94 (4.3)	5/44 (11.4)	0.129	2/30 (6.7)	0.605
*Enterococcus spp.*	6/94 (6.4)	3/44 (6.8)	0.923	2/30 (6.7)	0.956
*Anaerobes*	5/94 (5.3)	3/44 (6.8)	0.729	0/30 (0)	0.092
*M. tuberculosis complex*	4/94 (4.3)	1/44 (2.3)		1/30 (3.3)	
*Non-tuberculous mycobacteria*	1/94 (1.1)	0/44 (0)	-	0/30 (0)	-
*Cutibacterium spp.*	8/94 (8.5)	0/44 (0)	0.012*	1/30 (3.3)	0.304
*Candida species*	0/94 (0)	2/44 (4.5)	0.031*	0/30 (0)	-
*Aspergillus fumigatus*	0/94 (0)	1/44 (2.3)		0/30 (0)	-
*Corynebacterium spp.* *.*	1/94 (1.1)	0/44 (0)	0.380	0/30 (0)	0.456
**Previous Procedures**					
Previous procedures	58/136 (42.6)	13/56 (23.2)	0.011*	9/47 (19.2)	0.003*
Previous injection therapy	21/136 (15.4)	9/56 (16.1)	0.913	3/47 (6.4)	0.113
Previous surgery	37/136 (27.2)	4/56 (7.1)	0.002*	6/47 (12.8)	0.044*
**Surgical therapy**					
Surgical therapy	119/138 (86.2)	50/56 (89.3)	0.565	43/47 (91.5)	0.345
Time until surgery (days)	3.0 [2.0–6.0]	4.0 [2.0–8.0]	0.059	8.0 [4.0–14.0]	0.001*
**Outcome**					
Duration of inpatient stay (days)	25.0 [18.0–36.0]	32.0 [19.3–42.0]	0.046*	34.0 [28.0–50.0]	0.001*
1-year mortality	7/123 (5.7)	12/54 (22.2)	0.001*	19/42 (45.2)	0.001*
Recurrence	11/138 (8.0)	2/56 (3.6)	0.240	0/47 (0)	0.010*
Death or Recurrence within one year	18/123 (14.6)	14/54 (25.9)	0.072	19/42 (45.2)	0.001*
Days until death	98.0 [24.0—212.0]	69.5 33.0—130.8]	0.592	56.0 [38.0—126.0]	0.461

Data presented as number (percent), mean ± standard deviation or median [IQR], respectively. *BMI* body mass index; *CCI* Charlson comorbidity index; *ASA* American Society of Anaesthesiologists; *COPD* chronic obstructive pulmonary disease; *IBD* inflammatory bowel disease; *IE* infectious endocarditis; *CRP* C-reactive protein; *GFR* glomerular filtration rate; *Hb* haemoglobin; *CoNS*, coagulase-negative staphylococci; spp., species; *IQR* interquartile range, *p-value ≤ 0.05; p-values refer to the comparison of ONCO patients vs. CONTROL patients and CKD patients vs. CONTROL patients. All p-values reported are 2-sided

Psoas abscess and empyema occurred with a similar frequency in every group. In contrast, a neurological deficit occurred significantly more often in the CKD group (31.9%, P = 0.025). IE could be identified in 8.5% of CKD patients, 3.6% of ONCO patients and 2.9% of CONTROL patients. The presence of IE did not differ significantly between the groups. In the ONCO as well as in the CKD group, previous procedures prior to the diagnosis of VO were significantly less common than in the CONTROL group (ONCO: 23.2% vs. 42.6%, P = 0.011; CKD: 19.2% vs. 42.6%, P = 0.003). In the CONTROL group, 15.4% of VO patients underwent previous injection therapy and 27.2% of VO patients underwent previous surgery. Surgical treatment was required in approximately 90% of each group with no differences between the groups.

### Outcome

In the ONCO group, treatment failure was primarily due to death. 22.2% (12/54) of patients died within one year after diagnosis of VO which was significantly more than in the CONTROL group (5.7%, P = 0.001). Taken together with a recurrence rate of 3.6% (2/56), every fourth patient (26%) suffered from treatment failure in the ONCO group. Compared to the treatment failure of the CONTROL group of 14.6%, the difference did not reach significance (P = 0.072). In the CKD group, none of the patients suffered from recurrence of VO. Therefore, treatment failure in the CKD group was only defined by death within one year which was as high as 45.2% (19/42, P = 0.001).

**Fig. 2:**
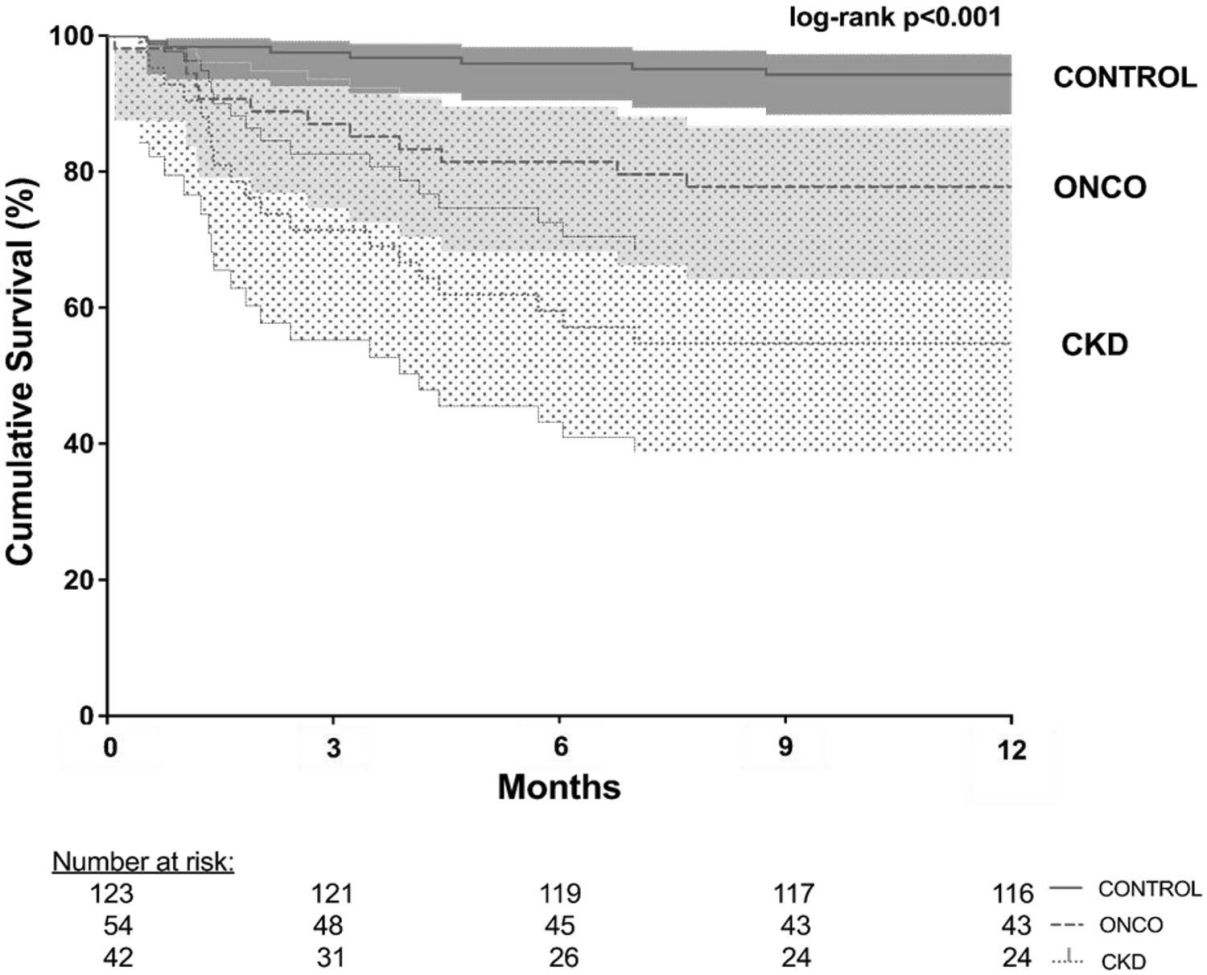
Kaplan–Meier curve. The Kaplan–Meier curve displays 1-year survival of CONTROL, ONCO and CKD patients within the first year after diagnosis of VO

Figure 2 shows 1-year survival of CONTROL, ONCO and CKD patients within the first year after diagnosis of VO. VO CKD patients had the highest mortality rate. After one year, the cumulative survival rate of VO CKD patients was only 54.8%. In the VO ONCO group, 77.8% of patients were still alive after one year. In the CONTROL group, 94.3% of patients were still alive.

Results of the multivariable analysis of independent predictors associated with treatment failure are shown in Table 2. In the ONCO group, the detection of *S.aureus* as underlying pathogen could be identified as an independent risk factor for treatment failure. Moreover, the presence of IE in the CONTROL group as well as the presence of bacteraemia in the CKD group, were significantly associated with a higher risk of treatment failure, respectively.

**Table 2:** Multivariable analysis of independent predictors associated with treatment failure (1-year mortality and/or recurrence of the disease) of VO patients without comorbidities (CONTROL), VO patients with an underlying malignancy (ONCO) and VO patients with CKD

	CONTROL n = 138 OR (95% CI), P value	ONCO n = 56, OR (95% CI), P value	CKD n = 47, OR (95% CI), P value
Infectious endocarditis	6.200 (3.594—10.696) 0.001*		
*S.aureus*		4.600 (1.749—12.100), 0.002*	
Bacteraemia			3.400 (1.254—9.216), 0.016*

*CI* confidence interval; *OR* odds ratio, *p-value ≤ 0.05

### Subgroup analysis

In order to take a closer look at the patients with malignant diseases, we performed a subgroup analysis (Table 3). We distinguished between solid and haematologic as well as active and inactive malignancies. An active malignant disease was defined as the diagnosis, treatment, recurrence, progression or metastasis of a malignancy within two years prior to the diagnosis of VO. Of the 56 oncological patients included in the subgroup analysis, two were lost to follow up. 43 patients were diagnosed with solid tumours, 8 patients were diagnosed with haematologic malignancies and 3 patients were diagnosed with both. 35 were classified as active and 19 as inactive malignancies. Logistic regression was performed to investigate risk factors for treatment failure. In the univariable analysis, the entity of the malignancy showed no significant correlation with treatment failure (P = 0.402) and was therefore not tested further in a multivariable analysis. Regarding the activity of the malignancy, the univariable analysis showed a significant influence of the activity of the malignant disease on treatment failure. Nevertheless, this significance could not be reproduced in the multivariable analysis. When using the unpaired t-test to compare active and inactive malignancies, patients with an active oncological disease showed a significantly higher rate of treatment failure (37.1% vs. 5.3%, P = 0.005).

**Table 3:** Subgroup analysis. Influence of entity or activity of the malignant disease

	Treatment failure, n (%)	*P*
**Entity of malignant disease**		
Solid (n = 43)	11 (25.6%)	0.402
Haematologic (n = 8)	2 (25.0%)	
Both entities (n = 3)	1 (33.3%)	
**Activity of malignant disease**		
Active (n = 35)	13 (37.1%)	0.005*
I nactive (n = 19)	1 (5.3%)	

*Active* is defined as diagnosis, treatment, recurrence, or progress of the malignant disease within the preceding two years before diagnosis of VO or metastasized malignancy, *p-value ≤ 0.05

## Discussion

To the best of our knowledge, this study is the first one to analyse treatment failure of patients with VO and an underlying malignancy. We present a comprehensive analysis of VO patients with an underlying malignancy and VO patients with CKD compared to VO patients without comorbidities, focusing on clinical characteristics, causing pathogens, outcome, and risk factors for treatment failure within the first year after diagnosis of VO. Our most important and significant results were the following: (i) Every fourth VO patient (26%) with a malignancy suffered from treatment failure within the first year after diagnosis of VO. (ii) VO patients with CKD showed a strikingly high treatment failure of 45%. (iii) The distribution of VO-causing pathogens in patients with a malignancy differed explicitly from the other groups. CoNS (27.3%) were the most common causative bacteria in the ONCO group, followed by *S.aureus* (22.7%) and gram-negative species (15.9%). (iiii) *S.aureus* as underlying bacteria in VO patients with a malignancy, the presence of bacteraemia in VO patients with CKD and the presence of IE in VO patients without comorbidities could be identified as independent risk factors for treatment failure, respectively.

Clinical characteristics of VO patients in our cohort did not differ from VO patients in other studies [9]. Since comorbidities are known to be associated with high mortality in patients with VO [12, 16], the high CCI score of the ONCO patients (score ≥ 5 in 68% of cases) and CKD patients (score ≥ 5 in 75% of cases) in our cohort might be one factor contributing to the high treatment failure. In the ONCO group, diabetes, heart failure and COPD were the most common comorbidities but each occurred in less than 20% of patients. In contrast, almost half of the CKD patients had a secondary diagnosis of diabetes and heart failure. This can most likely be explained by the fact that CKD and heart failure are common long-term consequences of diabetes. The high co-occurrence of diabetes and heart failure in patients with CKD may have significantly contributed to the high treatment failure rate in this population. Diabetes increases the risk of mortality and leads to poor prognostic outcomes, while congestive heart failure is associated with a significantly higher mortality rate. [19, 21]. VO patients of the CONTROL group were mainly categorized as mildly comorbid (≤ 2) by the CCI score. However, 42% of CONTROL patients were categorized as moderately comorbid which can be explained by age as a contributing factor. In the CCI score, people aged above 70 years are already classified as moderately comorbid.

*S.aureus* is known to be the most common pathogen causing VO in Europe [9, 22–26]. In addition, several studies have found that the prevalence of *S.aureus* is particularly high in CKD patients [20, 27–31]. This is in line with our findings. In our study, *S.aureus* was the most frequent causing pathogen of VO in the CKD and CONTROL group. *S.aureus* could be identified in more than half of the CKD patients (56.7%) as well as in 42% of the CONTROL group. Only CoNS were accounting for another relevant amount of approximately 20% of VO in the CKD as well as the CONTROL group. Apart from this, every other bacterial species was found in less than 10% of CKD and CONTROL patients.

In contrast to the CKD and CONTROL group, VO ONCO patients in our study showed a broad distribution of VO-causing pathogens: CoNS, *S.aureus*, *Streptococcus species* and gram-negative species each accounted for at least 10% of vertebral infections in the ONCO group. *S.aureus* was responsible for only 23% of VO in the ONCO group, while CoNS were the most frequently detected specimen in ONCO patients, accounting for 27% of cases. *Candida species* as underlying pathogens could exclusively be found in the ONCO group. The wide range of causative bacterial species in the patients with an underlying malignancy might be a result of immunosuppression and different infectious pathways. These include, in particular, endogenous infections, e.g. due to mucositis or enterocolitis, nosocomial acquired infections, especially venous catheter-associated infections, as well as community acquired infections with haematogenous spread. CoNS are part of the skin flora and frequently occur as causative pathogens following surgical procedures [32]. In addition, CoNS are frequent pathogens of vascular catheter-associated bacteraemia. Accordingly, CoNS were the most frequently found bacterial species in patients with a malignancy and a central venous catheter-related infection [33]. This is consistent with the high prevalence of CoNS in our ONCO group. ONCO patients are particularly at risk of infections caused by CoNS due to mucositis and the frequent use of central venous catheters for chemotherapy or in case of parenteral nutrition.

*Candida* species and gram-negative bacteria are part of the intestinal flora and can lead to bloodstream infections in oncological patients with mucositis or enterocolitis. In our study, gram-negative species were detected twice as often in the ONCO group as in the CKD and CONTROL group. The high incidence of gram-negative species in ONCO patients can be supported by the results of two retrospective studies from 2015 [34] as well as 2020 [29], which could find a significant association of the presence of gram-negative bacteria in VO patients with a malignancy. Overall, patients with malignancies show a higher rate of infections caused by gram-negative bacteria than by gram-positive bacteria [35]. With regard to vertebral infections, we were able to show that gram-positive pathogens were responsible for two thirds of VO in ONCO patients. Other risk factors for VO caused by *Candida spp.* are immunosuppression and the use of broad-spectrum antibiotics [36, 37]. Both risk factors are common in oncological patients. VO caused by *Candida* species is rare but accounts for an overall mortality of 15% [36].

To date, there are no studies which explicitly deal with VO patients with an underlying oncological disease that could be compared with our results. It is known from a large retrospective Japanese study including 7118 VO patients that malignant diseases are associated with a higher rate of in-hospital mortality (OR: 2.68; 95% CI 2.10–3.42) [16]. In our cohort, the risk of treatment failure in oncological patients was five times higher when *S.aureus* was detected as the causative pathogen (OR: 4.600; 95% CI 1.749–12.100; P = 0.002) although *S.aureus* was less common in ONCO VO patients than in the other groups. The presence of *S.aureus* bloodstream infection (SAB) is known to be associated with high mortality in VO patients in general as well as in oncological patients [12, 23, 38]. Regarding oncological patients, in a 14-year nationwide study in Denmark on SAB in haematological patients, a mortality rate of 44% was demonstrated [39]. A similar mortality rate of 41.2% (49/119) could be found in non-neutropenic cancer patients with SAB in a study from 2012 by Kang et al. [40]. These study results support our finding that *S.aureus* is an independent risk factor for treatment failure in oncological patients.

In the subgroup analysis of oncological VO patients, the entity of the malignancy did not show any significant association with treatment failure. An active malignancy was only significantly associated with treatment failure in the univariable analysis. This might be due to the small number of cases compared to the overall collective. Though we could not show that an active malignancy is an independent risk factor for treatment failure, we advise to reinvestigate this question in further studies.

Almost half of the CKD patients in our cohort died within one year after VO diagnosis which is highly concerning. The most commonly reported 1-year mortality rate in VO patients with CKD in literature is lower than in our cohort and accounts for approximately 20–25% of patients. In 2024, Ratiu et al. performed a comprehensive literature review and identified only 18 relevant studies on VO in haemodialysis (HD) patients which reveals the limited experience and sparse data available on VO patients undergoing HD [41]. In a systematic review from 2019 about VO with end-stage renal disease including 30 articles with 212 patients in total, the reported overall mortality was 24.1% [20]. In a study from 2017 by Lu et al. including 102 VO patients undergoing HD, 1-year mortality was 21.6% but there was an additional 20% of patients who suffered from recurrence within one year [27]. This result is comparable to the high treatment failure in our CKD group. However, in our cohort, recurrence did not occur in any of the CKD patients. Instead, every CKD patient with VO who suffered from treatment failure died. Bacteraemia increased the risk of treatment failure threefold in the CKD group regardless of the pathogen (OR: 3.400; 95% CI 1.254–9.216; P = 0.016). Bacteraemia is expected to cause kidney injury due to inflammation and ischemia which, as a result, leads to further decreased kidney function in CKD patients and therefore to more complications [21]. This highlights the importance of blood culture-samples, both as diagnostic tool and as prognostic factor.

### Strengths

To the best of our knowledge, this is the first study analysing VO patients with an underlying malignancy. As we compare different comorbidities, namely oncological patients versus CKD patients versus otherwise healthy patients, our study provides comprehensive information on a broad range of patients at risk of VO and their risk factors for treatment failure. Another strength is the 1-year-follow-up study design. By focusing not only on mortality but on treatment failure within one year after diagnosis of VO, our results add important information about the course of disease.

### Limitations

Because of the monocentric study design, our results might not be fully representative. Therefore, we advise to perform an additional prospective, multicentre cohort study to verify our findings. In addition, our data did not provide sufficient information about whether the events of death were clearly related to the infection itself, to the underlying disease or to other causes.

## Conclusions

In our study, every fourth patient (26%) with VO and an underlying malignant disease showed treatment failure within the first year after VO diagnosis. However, treatment failure of VO patients with CKD was almost twice as high (45%) as in the ONCO group. ONCO patients showed a broad distribution of VO causative pathogens which differed significantly from VO patients with CKD and VO patients without comorbidities. As there is currently no standardised recommendation for the empirical antibiotic treatment of VO patients , we particularly recommend to consider CoNS as causative bacteria when starting empirical antibiotic treatment in VO ONCO patients. Although *S.aureus* was less frequently found in ONCO patients than in the CONTROL and CKD group, *S.aureus* led to an almost five times higher risk of treatment failure in this group of patients. Therefore, oncological patients with VO caused by *S.aureus* should be monitored closely.

## Data Availability

Data available on request due to restrictions e.g. privacy or ethical. The data presented in this study are available on request from the corresponding author.
